# The use of nutrigenomics and nutritional biomarkers with standard care of long-term recurrent metastatic rectal cancer: a case report

**DOI:** 10.3389/fonc.2024.1451675

**Published:** 2024-12-02

**Authors:** Maree T. Brinkman, Sam Crofts, Hayden Green

**Affiliations:** Department of Clinical Studies and Nutritional Epidemiology, Nutrition Biomed Research Institute, Melbourne, VIC, Australia

**Keywords:** rectal cancer, recurrence, metastasis, folate, nutrigenomics

## Abstract

**Introduction:**

Distant metastases following standard treatment for locally advanced rectal cancer (LARC) are typically associated with poor disease-free survival. We report on a 52-year-old Australian male of Dutch ancestry with no family history of colorectal cancer or significant medical history who experienced bleeding per rectum for several months prior to a colonoscopy in July 2010. He was subsequently diagnosed with Stage IIb LARC.

**Case presentation:**

Despite treatment with curative intent, a distant recurrence to his left lung was detected in May 2012, upstaging him to Stage IV rectal cancer. He had repeated distant metastatic recurrences over the next 8 years, and treatment included multiple surgeries, chemotherapies, radiation treatments, a “watch and wait” period of 20 months, and personalised dietary management. Genetic and nutrigenomic testing identified that the case had *KRAS* and *MTHFR* mutations. As part of his dietary management, the case also had his levels of folate, vitamin B12, and vitamin D regularly monitored because of his genetic predisposition and history of deficiency for these key nutrients. Apart from changes in his CEA levels, sudden increases in the patient’s folate levels, inconsistent with dietary exposures preceded detection of each new distant recurrence, with significant decreases in the levels at the next follow-up measurement.

**Conclusion:**

A multimodal approach to this patient’s management appeared to contribute to his long-term survival of nearly 10 years from the initial diagnosis. Multidisciplinary management, including the use of additional biomarkers, may enhance survival rates in other similar cases with advanced disease resistant to differing therapies, and with potentially poor prognosis.

## Introduction

Over the past few decades, there has been an increasing trend in the number of younger cases diagnosed with advanced colorectal cancer (CRC) (1). Rectal cancer comprises around one-third of all CRC cases and is a distinct and heterogeneous disease presenting with different metabolic and genetic profiles, disease patterns, and responses to available treatments (2). While standard treatment for LARC, such as neoadjuvant combined chemotherapy (usually fluoropyridines) and radiotherapy (nCRT), along with surgery, has improved local disease control, distant metastatic recurrence reportedly occurs in approximately 30% of patients (3, 4). Distant metastasis is the leading cause of cancer-related death among LARC patients with a median survival between 24 to 36 months (5). The 5-year relative survival rate for Stage IV rectal cancer has been estimated at around 14% (6).

One of the biomarkers that have been associated with aggressive disease, poor treatment responses, and low overall survival rates is the Kirsten Rat Sarcoma viral oncogene homolog (*KRAS)* gene mutation (3, 7, 8). Epigenetic factors, such as DNA methylation patterns, are also reported to be associated with an increase in the number of younger patients with non-hereditary disease (9). Methylation is a dynamic process involved in regulation of gene expression by the addition of a methyl group to the 5-carbon position of the cytosine ring of DNA (10). DNA methylation can be modified by environmental factors, such as diet, with abnormal methylation patterns associated with cancer development and progression (10). Methylenetetrahydrofolate reductase (encoded by the *MTHFR* gene) is an enzyme involved in methylation and can influence DNA synthesis and repair (11). Genetic variations in the *MTHFR* gene have been associated with differing responses to treatments of rectal cancer as well as influencing folate metabolism and bioavailability (3, 11).

The effect of folate on colorectal carcinogenesis is complex and appears to have a “dual modulatory” role, with both high and low levels having been implicated in cancer risk, dependent on timing, dosing, and form of the nutrient (12, 13). This was evident from the varying responses to different folate levels reported in animal models (12). One animal study on rats found that folate deficiency led to a reduced risk of CRC (14), while another study on rats reported that low folate status increased the risk of CRC and that moderate increases in dietary folate were shown to be protective against developing the disease (13). It was also reported that no appreciable differences were observed at slightly higher folate doses (greater than 4 times the dose), while exceptionally high levels (1,000 times the dose) led to the development of neoplasms (13). Human intervention studies have investigated the effect of supplementation with folic acid (FA), the synthetic form of the nutrient for prevention of recurrent colorectal adenomas (CRAs) (12). One randomised clinical trial (RCT) allocated participants to either 1 mg/day of FA or a placebo for 3 years and found that this did not reduce the risk of CRAs (15). After an additional intervention and follow-up period, participants in the FA group had a higher incidence of advanced and multiple CRAs suggesting that FA supplementation may increase the risk of neoplasia (15). Two other RCTs using FA supplementation at 1 mg/day (16) and 0.5 mg/day (17) reported no effect on the risk of CRA recurrence or increased risk of advanced or multiple CRAs. The results from these RCTs highlight potential metabolic differences with FA, which may affect folate metabolism and biological pathways (12). Additionally, when administered at higher doses, FA, in some circumstances, may increase the risk of recurrence and disease progression, e.g., in participants with undetected microscopic CRAs (12).

While not all (18–20), several early epidemiological studies have reported inverse associations between folate levels and colorectal cancer (21–25). A large Danish cohort study reported that dietary folate had a protective effect against developing both colon and rectal cancer among those who consumed more than 10 g of alcohol per day (26). Variations in the potential effect of folate on CRC risk according to body site, gender, and genetic polymorphisms were also noted in a large Dutch study (27). In this study, dietary intakes of folate (pre folic acid fortification era) were associated with a decreased risk of rectal cancer but not colon cancer. Reduced risk was also observed for men but not women, and this association was most pronounced in those with *KRAS* mutated tumours (27).

We present a 52-year-old male of Dutch ancestry who was diagnosed with LARC and had nCRT, surgery, and adjuvant chemotherapy (aCT). He also had *KRAS* (G13D) and *MTHFR* (C667T rs1801133 and A198C rs1801131) mutations and had his first distant recurrence in his left lung within 2 years of initial diagnosis and treatment. Following this first distant recurrence, a nutritional assessment of the case identified potential nutritional deficiencies and metabolic issues based on nutrigenomics testing and a review of his medical history (vitamin D deficiency) and dietary intake, e.g., high alcohol and low folate intakes. From this point onward, he had personalised dietary management focussing on his folate, vitamin B12, and vitamin D levels, all of which were monitored and managed along with his standard medical care. The patient survived nearly 10 years, with eight of these years having multiple distant recurrences to both lungs, and later his liver.

## Case presentation

The patient was a 52-year-old white Australian businessman of Dutch ancestry. He had no family or personal history of cancer and had an unremarkable prior medical history consisting of irregular respiratory and viral infections, and, more recently, hypercholesterolaemia and vitamin D deficiency, which were managed by a statin (rosuvastatin 5 mg/day) and a supplement (intermittent use of OsteoVit D 1,000 IU/day), respectively. His daily alcohol consumption exceeded four standard drinks, and he was a past smoker of 10+ pack-years which he had ceased 30 years ago. At diagnosis (T3N0M0), the patient weighed 117 kg (BMI: 32 kg/m²) and was advised to lose 20 kg by his colorectal surgeon in preparation for the resection of his primary tumour to achieve good pathologic response and preservation of his anal sphincter/anus. Surgery was scheduled to follow neoadjuvant chemoradiotherapy (nCRT), using infusional 5-fluorouracil (5-FU), and radiotherapy (50.4 Grays). **Table 1** presents a summary of the multidisciplinary management of the patient over the 10-year history of his disease.

**Table 1:** Physical examinations, treatments, results, and responses over 10-year history of recurrent metastatic rectal cancer.

Date (Month/year)	Examinations and Results	Health status	Treatment and Responses
Jul – Sep 2010	• Rectal bleeding and altered bowel habits (from Feb 2010). • Colonoscopy and DRE - rectal adenocarcinoma in lower 3^rd^ 2cm above sphincters. • Weight: 117kg (BMI:32kg/m²)	• LARC Stage IIb – T3N0M0 • Managing daily activities (work and social) around TX schedule - ECOG Performance Status: 0	• nCRT infusional 5-fluorouracil (5-FU) & radiation (50.4 Gys) (Aug-Sep) • TX tolerated well - some mild peripheral neuropathy and loose bowels managed with Loperamide
Nov 2010	• MRI CT scan reduction in tumour size from nCRT. • Weight: 100kg (BMI:27kg/m²)	• Good recovery from surgery -ECOG Performance Status: 0	• Surgical resection of primary rectal tumour – temporary ileostomy (late Nov)
Dec 2010	• CT scan - LN clear & tumour appeared fully excised • Bloods: mild lymphopenia, ↓ vitamin D	• Patient going well and adapting to temporary stoma -ECOG Performance Status: 0	• No TX – lifestyle management • Osteo-Vit D 1,000IU
Jan - May 2011	• CT scans - no detectable disease • Rectal exam “nicely healed anastomosis” - No palpable lymphadenopathy • Bloods ↓ vitamin D, ↑ PSA (radiation)?, cell counts slightly down but able to continue TX	• Managed daily activities (work and social) around TX schedule - ECOG Performance Status: 0 • No longer on rosuvastatin	• aCT FOLFOX6 (8 cycles modified) commenced Jan and completed mid Apr 2011 • Some slight nausea, fatigue, peripheral neuropathy and cold sensitivity 1^st^ 48hrs • Stoma reversal (May) • Osteo-Vit D 1,000IU
Nov 2011	• CT scan - no evidence of disease recurrence • Colonoscopy and DRE normal • Bloods: within range - CEA 1.8 • Weight 105kg (BMI: 29kg/m²)	• Generally well and active - ECOG Performance Status: 0	• No TX – lifestyle management • CT scan and review in 6 months
May 2012	• CT scan - 1^st^ met L/lung 8mm diameter ovoid nodule lower left lobe • Bloods: CEA 2.2	• Generally well and active - ECOG Performance Status: 0	• No TX – lifestyle management • Follow-up CT scan in 3 months
Sep - Nov 2012	• CT scan - ↑ tumour to 14mm • PET scan - intense uptake of FDG in tumour	• Excellent recovery - ECOG Performance Status: 0	• Tumour resected (Oct) • Lifestyle management (some anorectal physiotherapy)
Aug 2013	• CT scan - small nodule detected in right lung • Bloods: CEA 2.1, ↓ vitamin D	• ECOG Performance Status: 0	• No TX - lifestyle management only
Nov 2013	• CT scan & PET scan - solitary nodule confirmed 2^nd^ Met/R Lung in right lower lobe • Bloods: CEA 3.6 ↓ WCC ↑Bili	• ECOG Performance Status: 0 • Viral URTI week prior to surgery in Dec	• Tumour resected (Dec)
Jun-Jul 2014	• CT scan - small left basal nodule at the margin of previous resection scar • Bloods: CEA 8 • 3kg weight gain	• Generally Well and Active - ECOG Performance Status: 0	• No TX – lifestyle management • CT scan and follow-up in 3 months
Aug - Sep 2014	• CT scan & PET scan confirmed Met in the left lower lobe and one 20mm in the left- sided mediastinum between his 9^th^ and 10^th^ ribs	• ECOG Performance Status: 0 • Viral URTI (Sep)	• No TX – lifestyle management • Osteo-Vit D 1,000IU
Oct - Dec 2014	• CT scan (Oct) • Bloods: ↑ CRP, ↓ vit D	• Good recovery – ECOG Performance Status: 0	• Lung and chest wall (mediastinum) resections (Oct) • 4-week conventional radiotherapy to chest wall (Dec)
Jan - Feb 2015	• Bloods: CEA 3.7 Feb, ↓ WCC ↓ lymphocytes • Lost 3kg after 1^st^ cycle of TX	• Working normal daily activities around TX - ECOG Performance Status: 0	• Chemotherapy - capecitabine, irinotecan + bevacizumab • Slight dose reduction of Irinotecan • Diarrhoea managed with Loperamide
Mar - Apr 2015	• Bloods: ↓ WCC, ↓ neutrophils, ↓ lymphocytes, ↓ Hb, ↓Hct, CEA 2.3 (late Apr) • Weight stable	• Working normal daily activities around TX - ECOG Performance Status: 0	• Chemotherapy - capecitabine, irinotecan + bevacizumab • Sore feet and hands, tiredness • Dose of capecitabine lowered (Apr) • Osteo-Vit D 1,000IU
May - Oct 2015	• CT scan - stable disease (May) • CT scan - no evidence of local recurrence or Met disease (Oct) • Bloods: ↓ WCC, ↓ neutrophils, ↑Bili, ↑albumin, CEA 2.2 (late Oct)	• Working normal daily activities around TX - ECOG Performance Status: 0	• Chemotherapy - capecitabine, irinotecan + bevacizumab • Maintenance regimen of chemotherapy (Aug) • Loose bowels managed with Loperamide • Osteo-Vit D 1,000IU
Nov - Dec 2015	• CT scan - a tiny left lower lobe nodule and small lingular nodule has appeared - small pulmonary Mets? • Bloods: CEA 2.6	• Working normal daily activities around TX - ECOG Performance Status: 0	• Maintenance capecitabine and bevacizumab (completed Dec) • Osteo-Vit D 1,000IU
Feb - Dec 2016	• CT scan - 2 new small Mets identified in right lung (Feb) • Bloods: CEA 1.2 (Sep), CEA 3.4 (Dec)	• Generally well and active - ECOG Performance Status: 0	• No TX - lifestyle management only • Regular medical monitoring • Osteo-Vit D 1,000IU
Jul - Dec 2017	• CT scan - 4 small bilateral lung tumours (Mets) detected with slight increase in size, small but stable with limited growth and no further Mets (Jul) • Bloods CEA 3.5 (Jul)	• ECOG Performance Status: 0 • Dental abscess • URTI • Shingles	• Stereotactic radiation to lungs (starts Aug) • Rest period (Sep) • Antibiotics for dental abscess • Tooth extraction • Antiviral for shingles
Jan - Feb 2018	• CT scan and PET scan - showed more lesions and larger left Met (Jan)	• Generally well and active - ECOG Performance Status: 0	• Stereotactic radiation (completed early Jan) • Lifestyle management
Jun 2018 – Jan 2019	• CT scan and MRI - new multiple liver Mets identified (Jun) • CT scan - disease stable (Dec) • Bloods: CEA 28.7 (Jul), CEA 2.34 (Dec), CEA 5.2 (Jan)	• Working normal daily activities around TX - ECOG Performance Status: 0	• Chemotherapy - capecitabine, irinotecan + bevacizumab (Jun – Dec) • Maintenance chemotherapy – capecitabine + bevacizumab (Jan)
Mar – Apr 2019	• CT scan - 1 tumour in lung, liver lesions persist (Mar) • Bloods: CEA 12.5 (Mar), CEA 17.8 (Apr)	• Working normal daily activities around TX - ECOG Performance Status: 0	• Maintenance chemotherapy - capecitabine + bevacizumab
May 2019	• MRI & CT scan - slight increase in liver Mets • Bloods: ↑ lipids, CEA 19	• Pancreatitis and duodenitis from SIRT leakage • Extremely unwell, GIT discomfort	• SIRT (Yttrium 90) – leakage of radioactive beads to duodenum and pancreas • Nexium 40mg and Dexamethasone 4mg post leakage
Jun 2019	• CT scan - disease progression (liver and lungs) • Bloods: CEA 28.8	• GIT pain • ↓ appetite & unintentional weight loss • Fatty foods problematic	• Chemotherapy - folfiri + bevacizumab • Nexium 40mg and Dexamethasone 4mg • Creon 10,000 with meals and snacks - not helping GIT pain
Jul - Oct 2019	• CT scans - disease progression with multiple new liver Mets (Jul) • CT scan - disease progression (Sep) • Bloods: ↑ CRP, ↑ LFTs, ↑ Glucose, ↑ B12, ↑ ESR, ↓Prot, ↓ Albumin, ↓ Globulin, ↓ WCC, ↓ neutrophils, ↓ lymphocytes, CEA 10.8 (Oct)	• No improvement in GIT discomfort • ↓ appetite, mood, activity, & engagement • Case in poor condition both physically and mentally	• Chemotherapy - folfiri + bevacizumab • Sepsis – hospitalised 1 week (Aug) • Chemotherapy ceases (Oct) • Nexium 40mg • Creon 10,000 not helping GIT pain – discontinued • Osteo-Vit D 1,000IU
Nov - Dec 2019	• Physical exam – develops jaundice, abdomen slightly distended (Dec) • Bloods: ↑ B12, ↑Bili, ↑LFTs, CEA 8.9	• Poor health condition - ongoing GIT discomfort, ↓ appetite, mobility, and nutritional status	• No TX – lifestyle management only • Nexium 40mg • Osteo-Vit D 1,000IU
Jan - Feb 2020	• CT scan – multiple hepatic Mets, no new Mets or progression (Jan) • Choledocholithiasis and liver abscess detected • Blood culture: infection (Enterococcus Faecium) • Bloods: ↑ Bili, ↑ LFTs, ↓Hb, ↓ Prot, ↓ Albumin, ↓ Na, ↑ CRP, ↑ Neutrophils, CEA 20	• Jaundiced • Cramping and nausea waves • Poor condition physically and mentally • Ascites	• Hospitalised - IV antibiotics (Amoxicillin & Vancomycin), ERCP, Biliary strictures stented (Feb) • Liver abscess aspirated. • Nexium 40mg • Osteo-Vit D 1,000IU
Mar – Apr 2020	• CT scan • Liver abscess • Bloods: ↑ LFTs, ↑ B12, ↓ Hb, ↓ Hct, ↓ Platelets, ↓ Albumin, ↑ CRP, CEA 81 • Erythema near right ankle	• Case at home with progressively lower food intake, reduced mobility and engagement • Case died at home (Apr)	• Ongoing intensive oral antibiotic treatment for liver abscess (Amoxicillin and Bactrim – not responsive) • Frusemide 40mg 1xbd and slow K 600mg 2xbd • Nexium 40mg • Osteo-Vit D 1,000IU • Bactroban 2% Ointment • Returned to hospital for draining tube to be inserted into liver abscess (Mar)

LARC, locally advanced rectal cancer; T3N0M0, Tumour 3 (size and extent of primary tumour), no cancer in lymph nodes (LN) and no metastatic spread of cancer; nCRT, neoadjuvant chemoradiotherapy; FOFLFOX, Leucovorin calcium (folinic acid), fluorouracil and oxaliplatin; aCT, adjuvant chemotherapy; TX, Treatment; Met/s, metastasis/metastases; Folfiri, chemotherapy regimen of leucovorin calcium, fluorouracil, and irinotecan; ↑ ↓, higher or lower than reference range; GIT, gastrointestinal; Gys, Grays; Bd, twice daily; Bili, bilirubin; CEA, carcinoembryonic antigen; CRP, C reactive protein; DRE, digital rectal examination; ESR, erythrocyte sedimentation rate; Hb, haemoglobin; Hct, hematocrit; IV, intravenous; LFTs, liver function tests; WCC, white cell count.

Due to the potential adverse side effects from nCRT (e.g., anaemia and neutropaenia) and the need to maintain his nutritional status for the upcoming surgery, dietary modification focussed on modest calorie restriction, mainly via a significant reduction in alcohol consumption and energy-dense “discretionary” foods. The patient modified his dietary intake from diagnosis and intentionally lost a total of 17 kg, weighing 100 kg (BMI: 27 m²/kg) by the time of his surgery approximately 4 months after his initial diagnosis.

The nCRT was effective in downsizing the rectal adenocarcinoma and downstaging his disease for surgery, which involved an open ultra-low anterior resection of his tumour with hand-sewn coloanal anastomosis with diverting loop ileostomy in late November 2010. Surgical resection of his tumour was successful, and the patient recovered well with no indication to require a long-term ileostomy. He was considered disease free at this stage.

Given the option, the patient chose to have adjuvant chemotherapy (aCT) with eight cycles of modified FOLFOX6 in early January 2011 and underwent an ileostomy reversal approximately 6 months later in May 2011. The patient returned to having regular/normal bowel function following some physiotherapy and dietary adjustment, e.g., an increase in soluble fibre and reduced lactose intake.

The patient kept well and was able to perform all daily activities (ECOG Performance status 0) and had three monthly medical follow-ups without any indication of disease recurrence. However, almost 2 years post initial diagnosis in May 2012, the first recurrence of distant disease was detected in the case’s left lung. No other lesions were identified anywhere at the time of his video-assisted thoracoscopic surgical (VATS) wedge resection to his left lower lung lesion in October 2012.

Initial genetic testing of the patient indicated that he had a *KRAS* (G13D) mutation, *NRAS/BRAF* wild type, and PD-L1 (IHC = 0%). Additional nutrigenomics testing was also conducted at diagnosis of the first distant recurrence to assist in optimising the patient’s nutritional and health status. This identified variations in several genes active in the one carbon and methylation pathway, including methylenetetrahydrofolate reductase (*MTHFR*—C667T and A1298C), 5-methyltetrahydrofolate-homocysteine methyltransferase reductase (*MTRR*), and Transcobalamin 2 (*TCN2*) genetic variants, which are also involved with folate and vitamin B12 metabolism and adequacy (10). Based on these results, the patient was genetically predisposed to reduced folate metabolism, lower vitamin B12 status, and higher homocysteine levels. From this point in his multidisciplinary management, the patient’s folate and vitamin B12 measures were monitored regularly along with other regular biomarkers such as his CEA, haematology, and biochemistry. The patient also had his vitamin D levels regularly checked because he had a history of vitamin D deficiency for several years prior to his initial diagnosis. This was the only supplement that he took across the course of his disease, and dosage was adjusted based on his blood levels and seasonal changes.

Given his excellent recovery (ECOG Performance Status 0), regular medical monitoring continued, but no further medical intervention followed this initial lung surgery. However, in August 2013, a second distant recurrence was detected in the patient’s right lung, and he had an excision of the right lower lobe tumour in December 2013. Regular medical follow-ups ensued, and in June 2014, another small nodule was detected in his left lung, which was confirmed as a metastatic tumour on a follow-up scan in September 2014, along with an additional moderately avid tumour undetected by CT scan but identified by PET scan in the case’s left-sided mediastinum between his 9th and 10th ribs. The tumour in the left side of his chest wall was consistent with the location of his drainage tube from the earlier resection in 2012 suggesting that some seeding had occurred. The patient underwent surgical resection of these new lesions in October 2014. Around this time, the patient was discovered to have a small liver cyst of 6 mm also detected on a CT scan.

Following the late 2014 resections, the patient underwent post-surgery radiation to his left-sided chest wall (50 Gy in 20 fractions) and chemotherapy for the first time in 4 years since his aCT in early 2011. The new chemotherapy regimen consisted of capecitabine, irinotecan, and bevacizumab from January 2015 until July 2015 with a lowered maintenance dose of capecitabine and bevacizumab only, until December 2015. A follow-up CT scan in early February 2016 identified two new small tumours in the patient’s right lung. He continued to have regular medical monitoring but was not prescribed any active medical treatment from January 2016 until August 2017.

**Figure 1:**
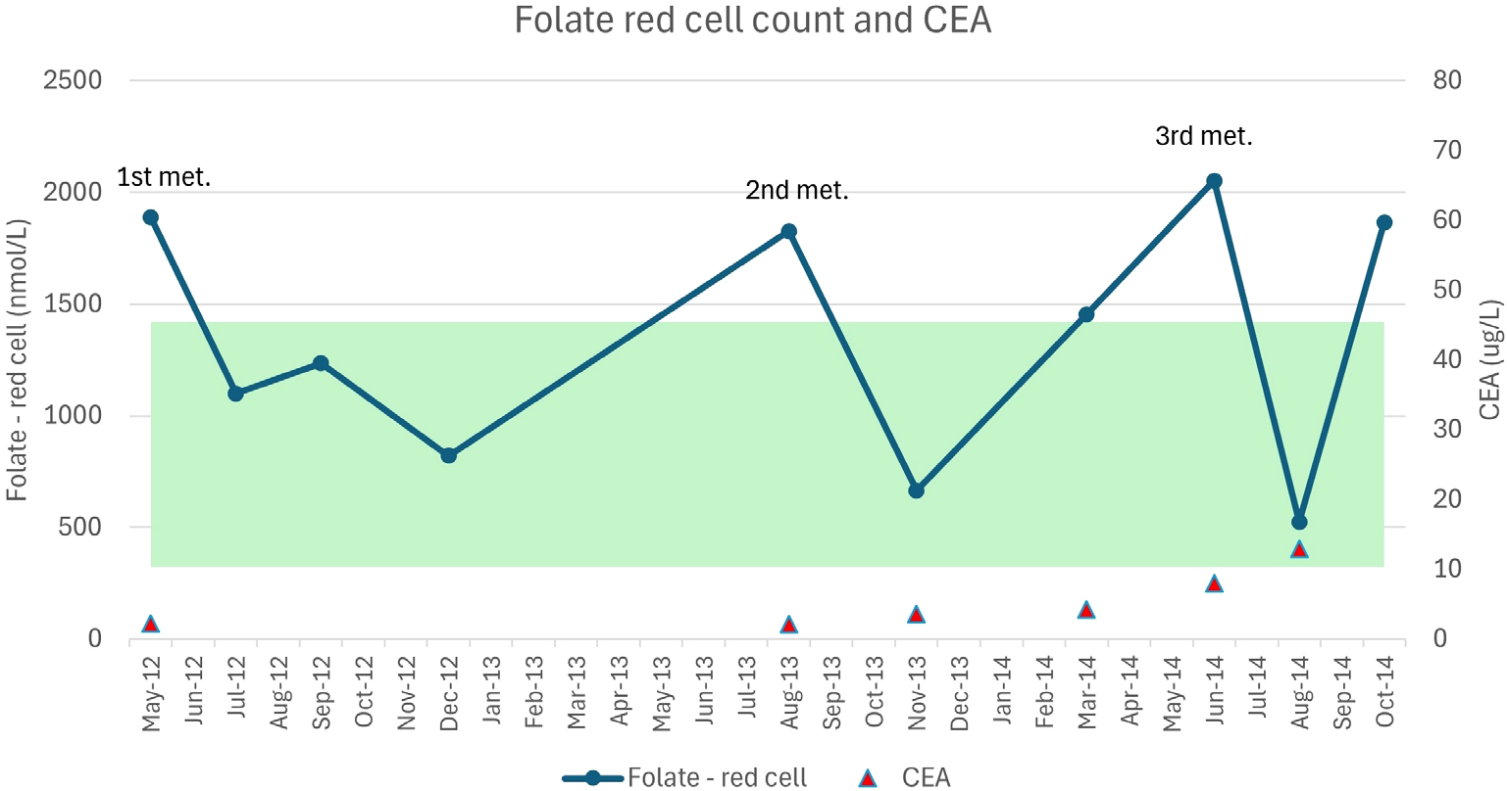
Red cell folate measures and the first three recurrent lung metastases. The image presents the changes in red cell folate levels against CEA (carcinoembryonic antigen) with detection of each new met (metastasis). The highlighted band depicts the standard reference range for red cell folate levels over this period.

During this “watch and wait” period, the patient’s health was exclusively managed via lifestyle interventions, predominantly by a personalised dietary plan. A pattern had emerged, as indicated in **Table 2**, whereby just prior to the detection of a new distant recurrence, the patient’s folate levels would rapidly increase, which was inconsistent with his dietary exposures that included, at the time, high alcohol and low folate intake, typically associated with low folate levels. This was directly followed by a significant decrease in folate levels. As the patient was not on any nutritional supplement that contained folate/folic acid, it was suggestive that the disease may be involved in these changes. This was evident at detection of the first recurrence, when the patient’s red cell folate levels were 1,888 nmol/L (CEA was 2.2 µg/L) and subsequently dropped to 1,100 nmol/L, which was also inconsistent with dietary exposures and in the absence of any medical treatment. Similarly, red cell folate was 1,826 nmol/L (CEA was 2.1 µg/L) at detection of the second lung metastasis and dropped to 665 nmol/L, with a corresponding increase in CEA to 3.6 µg/L. Red cell folate was 2,053 nmol/L (CEA was 8 µg/L) at detection of the third recurrence in the left lung and chest wall, with folate levels dropping further to 525 nmol/L and CEA increasing to 12.9 µg/L at follow-up measurements (see **Figure 1**).

**Table 2:** Folate, Vitamin B12, Vitamin D, and CEA levels from first distance recurrence of rectal cancer.

Date (Month/Year)	Folate	Vitamin B12	Vitamin D	CEA	Health Status / Management
May 2012	1888	353	48	2.2	1^st^ Met Left lung
Jul 2012	1100	459	50	N/A	No TX
Sep - Oct 2012	1235	255	72	N/A	↑ Met size - resection
Dec - Feb 2013	820	271	74	1.9	Well and active
Aug 2013	1826	443	46	2.1	2^nd^ Met Right Lung
Nov 2013	665	244	66	3.6	2^nd^ Met resected
Jun - Jul 2014	2053	272	123	8	Small nodule in Left lung
Aug - Sep 2014	525	242	68	12.9 (Sep)	Confirmed 3^rd^ Met + 1 Met in chest wall
Oct 2014	1867	289	114	N/A	Resections
Dec 2014	>54*	227	53	N/A	Radiation to chest wall
Jan - Feb 2015	29.8	228	70	1.8	Starts chemotherapy
Mar - Apr 2015	40.5	211	63	3	Chemotherapy
May -Jun 2015	34.8	266	69	1.6	Chemotherapy
Aug - Dec 2015	39.5	264	68	2.6	Maintenance chemotherapy to Dec
Mar 2016	26.8	225	87	N/A	Lifestyle management
May - Jul 2016	31	270	N/A	2.7	Lifestyle management
Sept 2016	18.6	294	89	1.2	Lifestyle management
Jul – Aug 2017	31.8	224	105	3.5	4 small bilateral lung Mets – Stereotactic radiation from Aug
Sep 2017	42.8	215	96	N/A	Lifestyle management
Dec 2017 – Jan 2018	22.5	199	134	N/A	Stereotactic radiation – Jan 2018
Jun - Jul 2018	27.6	N/A	113	28.7 (Jul)	Multiple liver Mets – starts chemotherapy
Dec 2018	34.7	262	115	2.34	Chemotherapy
Jan 2019	36.2	192	122	5.2	Maintenance chemotherapy
Mar 2019	26.4	229	108	12.5	Liver Mets + 1 Met in lung
May 2019	25.3	281	124	19	SIRT – leakage
Jun 2019	24.8	290	129	28.8	New chemotherapy
Oct 2019	33.9	>1476	78	10.8	Chemotherapy ceases
Nov 2019	15.9	1129	73	N/A	Poor health/condition
Dec 2019	21.6	819	90	8.9	Develops jaundice
Jan - Feb 2020	31.5	473	63	20	Liver abscess – Abs
Mar – Apr 2020	20	>1400	74	81	Health deteriorates – died Apr

Reference ranges: Folate (Red Blood Cell) = (>340nmol/L); Folate (Serum) (>9nmol/L); Vitamin B12 = (150-700pmol/L); Vitamin D = (>50nmol/L); Carcinoembryonic Antigen (CEA) = (>8.5ug/L); Abs = antibiotics; Mets = Metastasis/Metastases; N/A = Not available; * = folate changed from red blood cell to serum measures.

Over the 20-month monitoring phase, the patient focused on his nutritional and health status by maintaining a healthy weight and avoiding alcohol, which could adversely affect his folate levels. He followed a diet plan that consisted of adequate lean animal protein (~1 g/kg body weight—predominantly chicken and fish, with limited red meat and no processed meats) to maintain his cell counts, vitamin B12, and iron status. Plant foods comprised approximately 75% of his meal plan with a consistent high intake of leafy greens to ensure he met his daily requirements for folate. He also followed a personalised aerobic and weight training exercise program devised by exercise physiologists intermittently from late August 2016.

During the 20-month “watch and wait” period, the disease was kept relatively stable. A CT scan in July 2016 reported no increase in the number of metastases, just a slight increase in size but these were all still sub-centimetre. A scan of the chest, abdomen, and pelvis 12 months later in July 2017 detected four small bilateral lung tumours, with a slight increase in size of the largest ones to 12 and 15 mm. Given the slowly progressive nature of the disease, the multidisciplinary team decided that these tumours would be treated by stereotactic radiation. This was scheduled according to size, anatomical position, and avidity of the tumours in each lung. This involved 60 Gy in eight fractions over a 2.5-week course of stereotactic radiation to two metastases in his right lung in August 2017. Between this and his next course of treatment 8 weeks later, the patient suffered from a dental abscess and subsequent tooth extraction. In late October 2017, he had 55 Gy in five fractions to one of his left lung metastases. Prior to the final course of stereotactic ablative radiotherapy to his remaining left lung metastasis comprising 60 Gy in eight fractions from mid-December 2017 to early January 2018, a CT and PET scan showed two new mildly avid metastases to his right middle (6 mm) and right lower lobes (7 mm). Up until this point in his medical management, the case had maintained good overall health. He had tolerated all previous chemotherapies, radiotherapies, and surgeries well, requiring only minimal use of any analgesia, anti-emetics, and anti-diarrhoeal medications throughout the course of these different modalities. However, during this treatment period, he had not only experienced a severe dental infection but also a bout of shingles, and a prolonged upper respiratory infection requiring antibiotics suggesting that he was severely immunocompromised over this time.

In mid-2018 during regular monitoring of the patient’s blood, a rapid increase in CEA was observed, and follow-up investigations by CT and PET scans identified multiple metastases in the patient’s liver in June 2018. The patient recommenced a regimen of capecitabine, irinotecan, and bevacizumab in late June 2018 for 6 months proceeded by a maintenance dose of capecitabine and bevacizumab only until early May 2019. Some disease progression was detected via CT scan and MRI in early May 2019, and after considering stereotactic radiation to the liver, the multidisciplinary team opted for selective internal radiation therapy (SIRT) at the end of May 2019.

During the SIRT procedure, there was some leakage of radioactive microspheres (Yttrium 90) to the patient’s duodenum and pancreas. As a result, the patient suffered from pancreatitis and ongoing duodenitis and experienced unintentional weight loss (~10 kg), along with a compromised nutritional status and reduced quality of life. Follow-up blood work and imaging identified further disease progression, and the case commenced a chemotherapy regimen of FOLFIRI (5-FU, leucovorin, and irinotecan) and bevacizumab in late June 2019. He subsequently experienced an episode of sepsis from an unknown source and was hospitalised in late August/early September 2019. Chemotherapy ceased in October 2019, and the patient suffered from ongoing reduced appetite, mobility, and overall quality of life.

The patient became jaundiced in December 2019 and suffered from choledocholithiasis, complicated by *Enterococcus faecium* bacteraemia. The patient developed a liver abscess in February 2020, which did not respond to intensive intravenous and oral antibiotic treatment. Repeated insults to the liver, including progression of metastatic disease, caused the patient to develop significant ascites requiring regular drainage over the final few months of his life. He died in early April 2020, 9 years and 9 months after his initial diagnosis.

## Discussion

This case report demonstrates the benefit of a multidisciplinary approach to cancer management, including the assessment, monitoring, and management of key nutrients, folate, vitamin B12, and vitamin D in the patient’s ongoing battle with recurring rectal cancer. It also highlights tumour resistance to multimodalities and more aggressive forms of disease among some rectal cancer patients.

Although the scientific evidence is limited and equivocal at best for the role of folate in cancer prevention, progression, and survival, a particular pattern could be seen across the 8 years of monitoring this vitamin in our case. Sudden increases in the patient’s folate levels were observed, which were inconsistent with his dietary exposure at the time, e.g., when he had low dietary folate and high alcohol intake. Given that this dietary pattern should equate to low/suboptimal folate levels, it suggests that it may have reflected tumour metabolism rather than nutritional exposures.

We hypothesise that these elevated folate levels seen in the patient’s blood immediately preceding identification of a new distant recurrence possibly indicated hypermethylation and the silencing of tumour-suppressor genes (28). Although not statistically significant, van Engeland et al. reported that promoter hypermethylation was higher in colorectal cancers with low folate and high alcohol intakes (29). A mutation in the *MTHFR* (C677T) gene combined with a diet low in folate and high in alcohol has also been reported to increase the risk of colorectal cancer (30). This was a dietary pattern observed by this patient immediately preceding his initial diagnosis and subsequent first three distant metastatic recurrences. However, at the start of his 20-month “watch and wait” period, he commenced total abstinence from alcohol and followed a diet that was adequate in folate (mainly from leafy green vegetables), which coincided with fairly stable metastases and very limited growth over this period.

In terms of folate and DNA methylation, some studies have also conversely reported that low folate and high alcohol intakes are associated with global hypomethylation and increased risk of colorectal cancer (31). Sudden decreases in folate levels seen in our patient’s blood following the unexplained increases suggest possible hypomethylation resulting in chromosomal instability and further cancer development (10). Hypomethylation has also been associated with a higher proportion of colorectal cancers in younger patients with non-hereditary colorectal cancer, like our patient (9).

Based on our observations, both high and low folate levels appeared to reflect possible aberrant DNA methylation, and the combination of both hypermethylation and hypomethylation being involved in tumorigenesis and disease progression (32). A cross-sectional study of 189 colorectal cancer patients investigated the association between nutrients in the one-carbon metabolism pathway, particularly folate, along with a folate metabolism genetic polymorphism (*MTHFR*) and global DNA methylation in colorectal cancer (33). The authors of this cross-sectional study reported that serum folate levels were positively correlated with total dietary folate intake and global DNA methylation in the patients’ blood, and these measures influenced clinicopathological staging of disease (33).

While folate has a major role in DNA methylation, synthesis, and repair, and polymorphisms in the *MTHFR* gene involved in folate metabolism are reported to be predictive of cancer treatment outcomes (3, 11), the scientific evidence for optimal folate levels is critically lacking. Furthermore, the measurement of serum folate measures is not currently part of the standard range of blood tests for rectal cancer (34). Blood concentrations of folate can be affected by genetic predisposition, dietary exposure, food fortification, cancer treatments, and disease (3, 11, 28).

Another important consideration for monitoring folate status is the fact that wheat flour has been fortified with folic acid, a synthetic form of folate, for the past 15 years in Australia and longer in other parts of the world (35). While folate deficiency is relatively rare in the post fortification era, it is worth noting that both high and low levels of folate have been associated with risk of rectal cancer, and these levels are not currently being routinely checked in any population, including colorectal cancer patients (11). It was only by taking regular measures and tracking for trends that we could observe the unexpected significant changes in our patient’s folate levels across the course of his disease and modify his diet accordingly.

Recent studies have reported a positive association between vitamin D levels and colorectal cancer survival rates (36, 37). This patient had a history of low vitamin D levels for several years prior to diagnosis. Even though the patient continued taking his daily vitamin D supplement, his lower vitamin D levels toward the end of his life were most likely due to his impaired liver function, just like the elevation seen in his vitamin B12 levels at this time.

While findings from our study regarding folate levels is novel and warrants further investigation, a major limitation of this case report is that it only contains data collected from one case making it more hypothesis generating and unable to provide statistical validation. Also, as nutritional assessment and management were only instigated after detection of the first metastatic recurrence in 2012, nutritional biomarkers were unavailable earlier, i.e., prior to and at diagnosis. Reporter bias is another potential limitation; however, this should have been minimised by reporting data from different independent and objective sources including multiple blood measures of key nutrients of interest, which we could cross-check with nutrigenomic test results, dietary exposures, laboratory investigations, and medical reports over 8 of the 10 years of the patient’s history of disease.

As an essential nutrient involved in multiple molecular pathways, this case report demonstrates the potential advantages of including folate assessment in the multidisciplinary management of rectal cancer patients. Future research should be directed towards identifying those who may benefit from higher or lower levels of folate via a transdisciplinary approach and include nutrigenomics and folate status in large molecular/genomic, epidemiological, and clinical studies (38).

## Conclusion

In the age of precision medicine, nutritional profiling and management of certain subsets of rectal cancer patients, such as those with aggressive disease resistant to standard treatments, have potential to be an effective, low cost, and non-invasive addition to the multidisciplinary management of these patients. The use of nutrigenomic testing to identify potential metabolic issues and nutritional deficiencies is an area worthy of further investigation for its possible translation into evidenced-based clinical practice. Larger follow-up studies are essential to determine the role of folate status and metabolism in methylation, and carcinogenesis, and to confirm if regular monitoring and management of folate levels may be clinically beneficial to help improve patient outcomes and survival rates of rectal cancer (39).

## Data Availability

The raw data supporting the conclusions of this article will be made available by the authors, without undue reservation. Requests to access the datasets should be directed to maree.brinkman@nbri.com.au.
